# Integrating long-acting reversible contraceptives into primary care internal medicine practices: A clinical innovation to reduce wait time

**DOI:** 10.1177/17455057231219569

**Published:** 2023-12-21

**Authors:** Sheila M Quinn, Rebecca Goldfarb Terry, Shelby Boock, Judy A Shea

**Affiliations:** 1 Division of General Internal Medicine, Perelman School of Medicine, University of Pennsylvania, Philadelphia, PA, USA; 2 School of Nursing, University of Pennsylvania, Philadelphia, PA, USA; 3 Philadelphia College of Osteopathic Medicine, Philadelphia, PA, USA

**Keywords:** autonomy, contraception, menstrual health, primary care

## Abstract

**Background:**

Long-acting reversible contraception (LARC) has long been regarded as highly effective and safe. However, access is limited and lengthy when specialty referrals are required.

**Objectives:**

To integrate LARC services into an urban internal medicine primary care practice to decrease wait time for LARC procedures.

**Design/methods:**

This pre–post with control group study took place at two large urban academic primary care practices (Practices A and B) and included patients ages 18 to 45 years assigned female sex at birth. Pre-implementation baseline data were collected retrospectively from 2019 to 2020 by identifying subjects who requested LARC insertion or removal via their primary care practice and were referred to Obstetrics and Gynecology (Ob/Gyn) for the procedure. Wait time was noted from time of initial request in the medical record to time of procedure. Practice A developed an integrated primary care LARC program in which one of their LARC-trained providers began offering these procedures within their own practice. All other providers within the practice were educated on how to counsel patients about the devices and procedures. Practice B did not have an in-house LARC provider and continued referring patients to Ob/Gyn. Post-implementation data were collected prospectively 2021–2022.

**Results:**

Ninety-one patients in Practice A experienced a significant decrease in wait time (87 vs 21 days, *p* < 0.001) over the observation period, with a majority undergoing procedures on their first visit with the in-house LARC provider. Wait time for the 54 patients in Practice B remained unchanged (57 vs 47 days, *p* = .59), often requiring multiple specialty visits.

**Conclusion:**

Integrating LARC services into a primary care internal medicine practice can significantly reduce wait times for these procedures with the potential to contribute to increased reproductive and menstrual autonomy.

## Introduction

### background

Long-acting reversible contraception (LARC) has long been regarded as a highly effective and safe method for contraception and a widely-used method for menstrual management with minimal contraindications.^1^ Its use in the United States has increased over the past two decades, from 1.5% in 2002, to 10.3% in 2017.^2^ Historically, these devices which include intrauterine devices (IUDs) and the subdermal implant Nexplanon® have largely been managed in obstetrics and gynecology (Ob/Gyn) offices and some family medicine practices. As demand increases, some efforts have been made to incorporate LARC services into internal medicine primary care offices as well.^3–5^ In fact, two leading Internal Medicine professional societies endorsed the inclusion of LARC training for internal medicine residents in recent position publications.^6^,^7^ Integration into primary care has the potential to promote patient’s reproductive and menstrual autonomy by providing more rapid and direct access to LARC services. It can also potentially conserve resources by decreasing the number of visits and referrals needed per procedure. Existing research has laid preliminary groundwork describing practice-level feasibility, provider interest, and patient preferences.^3–5^,^8^

However, little has been done to examine how integrating LARC service within one’s primary care home versus referral to specialty providers influences wait time—a factor that not only greatly impacts patient care, but also may help inform a practice if it is worth the time investment to begin offering LARC in their practice versus relying on traditional referral routes. For example, in our institution, wait time for a new patient appointment in Gynecology can range from 2 to 8 weeks on the shorter end, often extending to several months. Lengthy waits for LARC can result in unintended or mistimed pregnancies, as well as perpetuate unwanted, distressing and at times dangerous menstrual patterns including dysmenorrhea, heavy bleeding and some catamenial syndromes. Likewise, lengthy waits for LARC removal can delay return to fertility for those trying to conceive, or timely device exchanges for those looking to continue safe and effective contraception. Given the many barriers that patients face in identifying and accessing qualified LARC providers, having these services available in one’s primary care internal medicine home could help decrease time to the desired procedure.

This clinical innovation and the observational analysis thereof describe a care model in which LARC services are integrated into an urban academic internal medicine primary care practice with the aim to decrease wait time for LARC procedures.

## Methods

### Design, setting, and participants

This clinical program and observational pre–post with control evaluation took place at two large urban academic primary care practices and included patients ages 18–45 years assigned female sex at birth. Table 1 includes detailed demographics of the two practices at baseline, defined as an 18-month period from January 2019 to June 2020.

**Table 1.** Baseline patient demographics for participating practices (January 2019 to June 2020).

	Practice A	Practice B
	*N* = 3152	*N* = 3001
*Age group*		
18–24	359 (11.4 %)	331 (11.0%)
25–34	1361 (43.2%)	1356 (45.2%)
35–45	1432 (45.4%)	1314 (43.8%)
*Race* *		
Black	1740 (55.2%)	1405 (46.8%)
White	1037 (32.9%)	1133 (37.8%)
Asian	183 (5.8%)	268 (8.9%)
Other, Unknown, Declined	192 (6.1%)	195 (6.5)
*Ethnicity*		
Hispanic/LatinX	141 (4.5%)	149 (4.9%)
Non-Hispanic/NonLatinX	2976 (94.1%)	2812 (93.7%)
Other, Unknown, Declined	35 (1.1%)	40 (1.3%)
Insurance		
Medicaid (%)	599 (19%)	510 (17%)

* *p* < 0.05.

### Program description and evaluation

**Figure 1.**
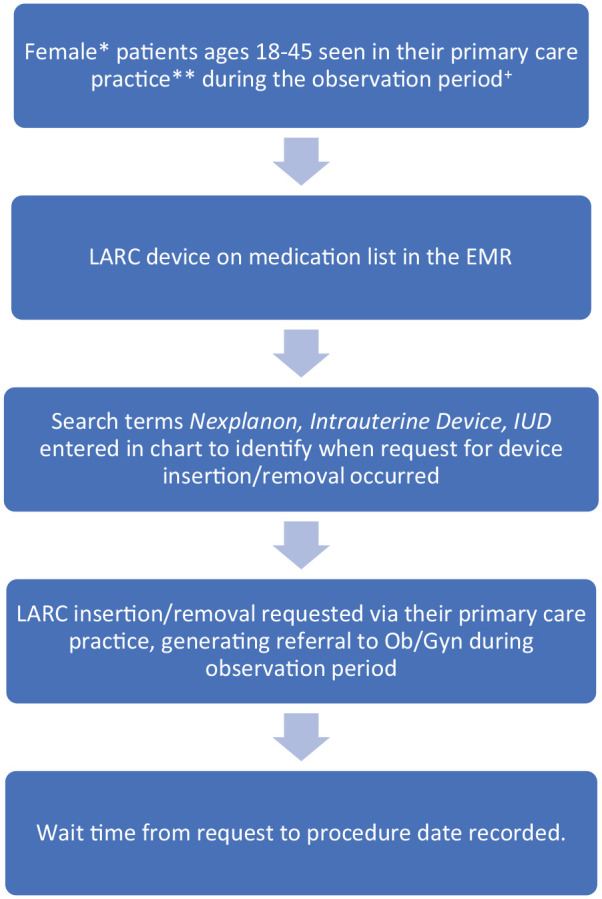
Subject identification process. *Female sex identified at birth. **This same identification process was followed for both Practices A and B. ^+^Pre-implementation observation period: January 1, 2019, to June 30, 2020. Post-implementation observation period: January 1, 2021, to June 30, 2022.

Pre-implementation baseline data were collected retrospectively from January 2019 to June 2020 by identifying subjects who requested LARC insertion or removal via their primary care practice and were referred to Ob/Gyn for the procedure. This was accomplished by first identifying all charts meeting demographic data and having one of these devices listed on their medication list during the pre-implementation observational period. Then, electronic medical record search terms including *Nexplanon*, *Intrauterine Device*, and *IUD*, were entered to extract qualifying encounters (in which the patient requested insertion or removal of the devices via an encounter with their primary care provider’s office). Qualifying charts were then reviewed to ascertain wait time, in days, from first mention of the desired procedure recorded in a clinical encounter, to the actual date of procedure being performed. A concise depiction of this subject selection process is shown in Figure 1. It was also noted if the patients had an established relationship with our health system’s Ob/Gyn practices (seen within the last 2 years). Although not a primary outcome, data were collected on prescription contraception use among the two practices at baseline for all subjects of the qualifying demographic.

Beginning in July 2020, Practice A developed an integrated primary care LARC program. This was possible because one of their physicians (S.Q.) underwent LARC training during an Adolescent Medicine fellowship and served as the sole LARC provider, offering these procedures one half day per week for the practice. All physicians and advanced practice providers at Practice A attended a formal didactic session delivered by the LARC provider. These sessions covered the available LARC devices, best-practices for counseling within a reproductive justice framework, how to refer patients to the in-practice LARC provider, as well as other routes of referral such as sending patients to Ob/Gyn at our institution or other family planning community sites. Resident physicians within Practice A also received similar didactics during their ambulatory educational conferences. Nurses at Practice A were educated about the LARC devices, including how to submit for insurance pre-certifications which was done by a designated licensed practical nurse at the time of patient request. They also learned which equipment was needed for each procedure so they could order and prepare supplies accordingly, and they were trained on how to assist for IUD insertions. Scheduling personnel were informed of the procedures that may be requested and necessary time slots. Signs describing the services offered were placed in all exam rooms in Practice A. Practice B did not have an in-house LARC provider and continued referring patients to Ob/Gyn. The same data described above (wait time and prescription contraception use) were collected post-implementation prospectively from January 2021 (when Practice A started placing and removing LARC devices) through June 2022 for both practices.

### Statistical analysis

Data on wait times were analyzed using comparisons of medians for independent samples. Differences in proportions between groups were compared with the chi-square statistic.

## Results

At Baseline, 24 patients in Practice A requested LARC procedures via their primary care practice and waited a median 81 days for their LARC procedure to be performed in the Ob/Gyn practice to which they had been referred. This does not include those who requested the procedure directly from Ob/Gyn (without going through their primary care office) or those who sought the procedure elsewhere in the community. Seventy-five percent of these 24 patients underwent their procedure at the first appointment with the Ob/Gyn that they saw. Thirty-three percent of these 24 patients were already established with an Ob/Gyn at the time of their appointment request. Twenty-seven patients in Practice B requested LARC procedures via their primary care practice and waited a median of 57 days for their procedure. Seventy-four percent underwent their procedure at the first appointment and 15% were already established with an Ob/Gyn at time of request.

In the 18 months following development of the integrated LARC program in Practice A, 91 patients requested LARC procedures via their primary care practice and waited a median of 21 days for their LARC procedure. The change from baseline to post-implementation was significant, *p* < 0.001; 91% of the patients underwent their procedure on the first appointment with the in-house LARC provider. In the same time period in Practice B, 54 patients requested LARC procedures via their primary care practice and waited a median of 47 days (*p* = 0.59 compared to baseline), with 52% of them undergoing their procedure at the first appointment with Ob/Gyn.

Although not a primary outcome, and not statistically compared, among patients using a form of prescription contraception during this time, we observed a small increase in the proportion of patients using LARC in Practice A once it was offered in-house (43% pre- vs 50% post-implementation) versus an unchanged proportion of patients in Practice B (49% vs 49%) who selected LARC over the observation period.

## Discussion

With continued increase in LARC demand nationally, challenges in access to such services are becoming more apparent.^9^,^10^ Among other factors, relying on referrals to specialists for these procedures potentially lengthens wait times, contributing to a potential increase in the number of appointments needed for a single procedure and a potential decrease in menstrual and reproductive autonomy for patients. Data from this clinical innovation show that offering LARC services in one’s primary care home significantly reduces wait times and increases the percentage of patients undergoing their requested service on the first visit with a LARC provider. Similar to studies in more rural settings, this clinical innovation is among the first to demonstrate that an integrated Primary Care LARC program could help reduce wait times for patients in an urban academic healthcare setting.^11^ With decreased wait times, comes quicker access to a patients’ chosen form of contraception or menstrual management and quicker access to device removals for those who no longer want the device. Some patients’ preferences to receive their gynecological care within their primary care provider’s office may be molded by past experiences with access, wait times, and cost associated with subspecialty care.^8^

Regarding the overall shift in preference for LARC compared to other forms of prescription contraception in Practice A over the study period, this is not dissimilar from previous studies where LARC devices were selected more often when certain barriers, namely financial, informational, and access were deconstructed.^12^ It also approximates the national proportion of people using prescription contraception who select LARC.^2^ It is not possible, within the context of this analysis, to know for sure if there were other factors that influenced this shift in patient preference in our clinical program over time. Ascertainment bias is a possibility, although more likely is that signage in the Practice A that informed patients of the services offered, likely prompting more conversations between patients and providers. Furthermore, providers likely felt more equipped to discuss the options given the didactics in which they participated.

This relative ease of this clinical program’s integration is also attributed to the fact that a provider in Practice A (SQ) was already trained in LARC upon arrival to the practice. To expand access to other internal medicine practices within our health system, additional providers are now opting to undergo LARC training from this index provider. A similar model could be considered in other systems where a primary care provider could undergo training with a willing Ob/Gyn or LARC-trained Family Medicine provider in order to expand access to non-complicated LARC insertions and removals.

We are hopeful that this clinical innovation can serve as a model for other primary care practices looking to integrate LARC services with an aim to minimize wait time and maximize patient-centered access to reproductive and menstrual care in the primary care setting. Qualitative inquiries could augment our understanding of how and why patients choose to have procedures at their primary care office vs Ob/Gyn and could offer further insight into outcomes not visible to us within our own health system (e.g. having procedures done at a community clinic). Qualitative work could also potentially explore patients’ experiences having these procedures done outside of an Ob/Gyn office, especially for those who have undergone procedures in both settings. It would be ideal to offer same-day LARC procedures to patients on the day of their request, instead of scheduling with the in-house LARC provider. This would require training more providers to provide these services, which is now underway at our institution. For device insertions, for patients using insurance other than Medicaid plans (which almost invariably cover new devices at 100% without prior authorizations), additional time would need to be built into the visit to verify coverage with private insurance plans prior to placing new devices.

The value of this clinical innovation is strengthened by the fact that it describes a relatively diverse population in terms of age, race, ethnicity, and Medicaid coverage. It is limited by the setting being a single urban academic institution. In addition, power analysis for sample size was not done given the focus of this study was largely quality improvement and program development. However, similar small studies have been done in rural areas, making this clinical innovation unique and additive to existing literature.^11^,^13^ Wait times were taken at face value– we did not consider the number of cancelations, no-shows, or reschedules that occurred between an initial request for a LARC procedure and the ultimate delivery of this service. It is possible that wait times were longer because of patients’ own preferences to postpone appointments. It is also possible that a patient preferred their own Ob/Gyn provider, if already established, or the idea of an Ob/Gyn performing the procedure over an internist, and thus opted for a longer wait. In addition, the post-implementation data were collected during the COVID-19 pandemic and may have inflated some wait times with Ob/Gyn. For example, if patients in practice B were referred to Ob/Gyn for their LARC procedure, the first visit may have taken place via telehealth where method counseling, device selection, and consent occurred, with the patient returning for a second visit for the actual procedure, potentially skewing the percent of patients undergoing same-day procedures after their Ob/Gyn referral. Overall, innovations such as this one are promising for increasing access to and autonomy surrounding one’s menstrual and reproductive health.

## Conclusion

Overall, this study shows that integrating LARC services into a primary care internal medicine practice can significantly reduce wait times for these procedures. This has the potential to contribute positively to increasing reproductive and menstrual autonomy as well as possibly conserving resources if a substantial volume of requests exists within the practice. Although this sample is small, it proves to be diverse regarding race, age, and Medicaid coverage and is additive to the current body of literature that is working to expand LARC access via primary care settings.

## Declarations

**Ethics approval and consent to participate:** This study was deemed exempt from the University of Pennsylvania Institutional Review Board, as it was deemed a Quality Improvement initiative, and did not meet the definition for human subjects’ research. There is no applicable consent for subjects.

**Consent for publication:** Not applicable.

**Availability of data and materials:** Not applicable.
